# Role of S-palmitoylation in digestive system diseases

**DOI:** 10.1038/s41420-025-02629-z

**Published:** 2025-07-18

**Authors:** Hanqing Li, Qiuxiang Yuan, Shuangshuang Wang, Tao Yu, Xingsi Qi

**Affiliations:** 1 Department of Gastroenterology, The People’s Hospital of Qingdao Chengyang, Qingdao, China; 2 Institute for Translational Medicine, The Affiliated Hospital of Qingdao University, Qingdao, China; 3 Department of Gastroenterology, The Affiliated Hospital of Qingdao University, Qingdao, China

**Keywords:** Post-translational modifications, Gastrointestinal diseases

## Abstract

Digestive system diseases, including liver diseases, gastrointestinal cancers, and inflammatory bowel diseases, pose major health challenges worldwide. These conditions are influenced by a range of key metabolic signaling pathways, many of which are regulated by palmitoylation. Palmitoylation is a type of lipid modification catalyzed by DHHC palmitoyl S-acyltransferases (DHHC-PTAs) and depalmitoylases, which play critical roles in modulating protein localization, stability, and signal transduction. Dysregulation of S-palmitoylation is closely associated with numerous diseases, including these of the digestive system, through multiple key processes such as immune responses, lipid metabolism, and cellular signaling. Decades of investigations have driven the development of a large body of inhibitors targeting zDHHCs and depalmitoylases, such as S-(2-acetamidoethyl) 2-bromohexadecanethioate (MY-D-4), Artemisinin and Lomitapide. This review provides a comprehensive summary of the role of palmitoylation in digestive system diseases, discusses its effect on disease mechanisms. By elucidating the regulatory functions of palmitoylation under these conditions, this review aimed to identify new strategies for the diagnosis and treatment of digestive system disorders.

## Facts

- Palmitoylation plays a critical role in modulating protein localization, stability, and signal transduction.
- Dysregulation of S-palmitoylation is closely associated with numerous diseases, including these of the digestive system.
- A large body of inhibitors targeting zDHHCs and depalmitoylases may the effective strategies for the treatment of digestive system disorders.

## Open Questions

- The reversible nature of palmitoylation complicates the precise detection and characterization of this modification in vivo. Traditional methods can identify and quantify palmitoylation states, but fail to capture real-time dynamic changes. How to solve this problem?
- The hydrophobic nature of palmitoylation makes it difficult to solubilize and purify the modified proteins, further complicating research.

## Introduction

The digestive system, which consists of the digestive tract and associated glands, is a continuous anatomical structure that plays crucial roles in swallowing, digestion of food, absorption of nutrients, and excretion of residual wastes [1]. The onset and progression of digestive system diseases occur over the long-term and are mediated by complex processes involving a combination of genetic mutations, inflammation, infections, and malignancies [2–5]. Over the past decades, advances in molecular biology, immunology, and pharmacogenomics have reshaped our understanding of the pathogenesis of these diseases, contributing to the development of increasingly targeted pharmacological interventions. For instance, the advent of monoclonal antibodies targeting tumor necrosis factor-alpha (TNF-α) or integrins has revolutionized inflammatory bowel diseases management, offering clinical remission for patients unresponsive to conventional therapy [6]. Similarly, the emergence of immune checkpoint inhibitors has opened new therapeutic avenues for subsets of gastrointestinal malignancies, including microsatellite instability-high (MSI-H) colorectal cancer [7]. Despite ongoing advances in treatment strategies, the morbidity and mortality rates associated with certain diseases have continued to increase annually, posing a significant threat to human health [8]. Consequently, investigating the fundamental pathophysiological causes of gastrointestinal disorders and identifying innovative and more efficacious treatment strategies has gained importance.

Palmitoylation was first identified in 1979, typically involves the attachment of a hydrophobic fatty acid (FA) chain that promotes the incorporation of proteins into the phospholipid bilayer of cellular membranes [9, 10]. This modification can affect the binding of the modified protein to the cellular membranes or intracellular membranes, alter the membrane localization and intracellular trafficking of palmitoylated proteins, influence protein-protein interactions, and ultimately regulate cellular signal transduction [11, 12]. Palmitoylation is classified into three types according to the modification site residues: S-palmitoylation, N-palmitoylation, and O-palmitoylation [13]. Most palmitoylation modifications occur at the cysteine (Cys) residues of proteins through a thioester bond, which is known as S-palmitoylation [13].

Accumulating dates have revealed that dysregulation of S-palmitoylation is closely related to various human diseases, including cancers [14], neurodegenerative diseases [15, 16], and digestive system diseases (e.g., inflammatory bowel diseases [17–20], metabolic dysfunction-associated steatotic liver disease [21–23], gastric cancer [24–26], pancreatic cancer [27–29], colorectal cancer [30–32], and hepatocellular cancer [33–35]). For instance, the palmitoylation of synaptic proteins (like VAMP2, SNAP25, and STX1) and postsynaptic proteins can affect synaptic transmission by regulating synaptic vesicle fusion, localization and clustering in neurons [15]. Additionally, the palmitoylation of lipid metabolism-associated proteins (such as CD36) can influence fatty acid uptake through alteration of its cell membrane translocation in the heart, liver and in tumors [20, 36, 37]. The palmitoylation-depalmitoylation cycle in the digestive system is crucial for several vital signaling pathways, including signal transducer and activator of transcription (STAT3), NLR family pyrin domain-containing 3 (NLRP3), and nucleotide-binding oligomerization domain-containing protein (NOD) 1/2 [18–20], as well as for immune processes such as programmed death-ligand 1 (PD-L1) degradation [38]. Consequently, a thorough comprehension of the palmitoylation of key proteins may be help elucidate the underlying processes and provide novel therapeutic strategies for digestive diseases.

This review sought to tackle this gap via providing a comprehensive investigation of the function of palmitoylation in digestive system diseases, focusing on its biochemical mechanisms, its impact on protein function and cellular processes, and its significance in disease pathogenesis. We also investigate the potential of targeting palmitoylation as a therapeutic approach for treating digestive system diseases and provide insights into future research directions and potential applications.

## Regulatory enzymes and physiological functions in s-palmitoylation

### S-palmitoylation and its regulatory enzymes

**Fig. 1:**
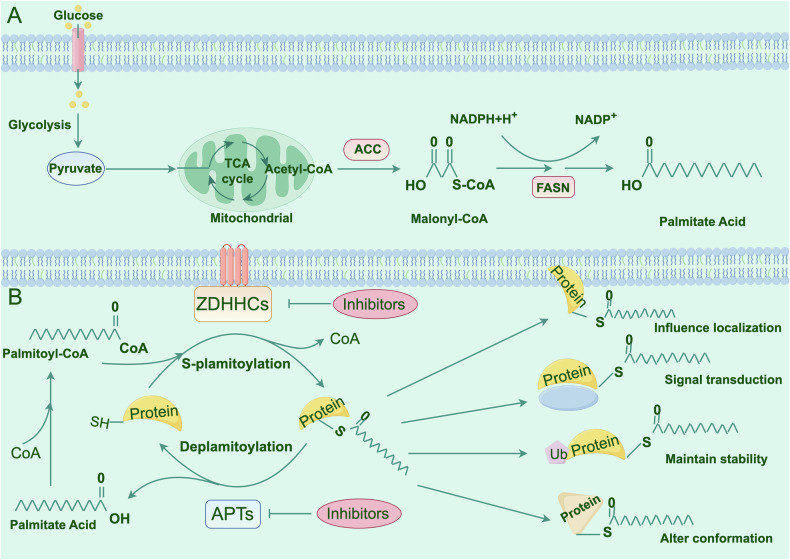
Dynamic regulation and physiological function of s-palmitoylation. **A** The progress of palmitic acid synthesis. Glucose undergoes glycolysis producing pyruvate, and the later entered mitochondrial producing malonyl-CoA via a series of reaction. Subsequently, FASN catalyzes the sequential reactions producing palmitic acid. **B** The regulation of palmitoylation-depalmitoylation.

Palmitic acid (PA) is a long-chain saturated FA, primarily originating from dietary sources and endogenous fatty acid metabolism [39]. It is synthesized from precursor FAs by fatty acid synthase (FASN) [40]. Initially, the glucose absorbed by hepatocytes undergoes glycolysis to produce pyruvate. Upon entering the mitochondria, pyruvate is converted to acetyl-coenzyme A (CoA) by pyruvate dehydrogenase (PDH). Acetyl-CoA carboxylase (ACC) then catalyzes the conversion of acetyl-CoA to malonyl-CoA. Subsequently, FASN catalyzes the sequential reactions that convert acetyl-CoA and malonyl-CoA to PA (Fig. 1A). Previous evidence has indicated that PA exerts a vital effect on anti-inflammatory, antioxidant, and immune-enhancing properties [41]. Additionally, PA is indispensable for palmitoylation. Palmitoylation is a reversible lipid modification characterized by the attachment of PA, a 16-carbon saturated FA, to Cys residues via thioester bonds. Palmitoylation significantly influences protein activity and facilitates membrane translocation, and it is catalyzed by the protein acyltransferase (PAT) family, also known as zinc-finger DHHC-type (ZDHHC) proteins [42].

DHHCs are multi-pass integral membrane proteins characterized by 4–6 transmembrane domains (TMDs) and a conserved aspartate-histidine-histidine-cysteine (DHHC) motif [43]. This sequence folds into a zinc finger domain and coordinates with zinc ions to stabilize the protein structure [44]. To date, 23 DHHC domain-containing proteins have been identified in mammals, all of which exhibit palmitoyl-transferase activity. Most of these enzymes are localized in the endoplasmic reticulum (ER) and Golgi apparatus, with a few found on the plasma membrane (PM) [45]. Previous investigations have indicated that protein palmitoylation often consists of two stages: in the first stage, the auto-acylation of ZDHHC, whereby palmitoyl-CoA interacts with the Cys in the DHHC motif, yields an acyl-enzyme intermediate and liberate free CoA-SH; in the next stage, the acyl group is conveyed to the Cys residue of the substrate protein, finalizing the palmitoylation process [46, 47] (Fig. 1B).

The reversibility of palmitoylation, a process mediated by acyl-protein thioesterases (APTs), which are also known as depalmitoylation enzymes, is crucial for its biological function. These enzymes hydrolyze thioester bonds, releasing PA and restoring the unmodified state of proteins. This process not only regulates the membrane association of proteins, but also potentially affects their degradation pathways. According to existing research, depalmitoylation enzymes are classified into three main categories: acyl-protein thioesterase 1/2 (APT1/2), palmitoyl-protein thioesterase-1/2 (PPT1/2), and α/β-hydrolase domain 17 (ABHD17A/B/C) and α/β-hydrolase domain 10 (ABHD10). Table 1 summarizes the localization and potential functions of these enzyme classes.

**Table 1:** Localization and function of zDHHCs and depalmitoylation enzymes.

zDHHCs	Localization	Targets	Function	Refs.
zDHHC1	ER, Golgi	IGF2BP1, Gpm6a	Reducing the stability of LIPG mRNA; regulating Procr protein stability; mediating signal transduction of MITA/STING.	[32, 148, 149]
zDHHC2	ER, Golgi	AGK, CKAP4, LCK	Regulating translocation of AGK into the PM and activating the PI3K/AKT/mTOR signaling pathway in ccRCC. Influencing CKAP4 localization to the PM.	[150, 151]
zDHHC3	Golgi	PD-L1, Cadm4 TLR9	Preserving PD-L1 from ubiquitination-mediated lysosomal degradation; influencing Cadm4 localization to the PM; altering the membrane localization of TLR9 in the Golgi.	[38, 152, 153]
zDHHC4	ER, Golgi, PM	GSK3β	Promoting the EZH2-STAT3 axis by GSK3β palmitoylation.	[154]
zDHHC5	PM	NOD1/2, CD36, FAK NLRP3 RIPK1	Altering NOD1/2 and CD36 localization to the PM; facilitating FAK transportation to the PM. Promoting NLRP3-NEK7 interaction and inflammasome activation. Promotes RIPK1 recruitment to complex I and promotes cell death.	[18, 52, 72, 100, 155]
zDHHC6	ER	AEG-1, PPARγ	Promoting HCC progression by regulating AEG-1 protein stability; regulating fatty acid synthesis by increasing PPARγ stabilization.	[30, 33]
zDHHC7	Golgi	STAT3, GSDMD	Facilitating STAT3 recruitment and phosphorylation at the cell membrane promoting GSDMS-NT translocation to the plasma membrane.	[19, 63, 101]
zDHHC8	Golgi	SLC7A11	Facilitating the stability of SLC7A11 by decreasing its ubiquitination level.	[156]
zDHHC9	ER, Golgi	GLUT1, Bip, β-catenin	Maintaining GLUT1 plasma membrane localization; enhancing Bip’s stability and maintaining its location inside the ER; promoting β-catenin’s ubiquitination and degradation.	[102, 157, 158]
zDHHC11	ER	ATGL	Regulating lipid droplet catabolism by modifying ATGL in hepatocyte cultures.	[159]
zDHHC12	ER, Golgi	CLDN3 NLRP3	Promoting the precise localization and stability of CLDN3 on the PM; augmenting Enhancing NLRP3 degradation through the chaperone-mediated autophagy pathway.	[160, 161]
zDHHC13	ER, Golgi	ULK1	Enhancing the phosphorylation of ATG14L and activating PI3-kinase signaling.	[162]
zDHHC14	ER, Golgi	GSDMD	Regulating GSDMD cytomembrane localization.	[163]
zDHHC15	Golgi	Nrp2	Regulating Nrp2’s localization and functioning in cortical neurons.	[164]
zDHHC16	ER	PCSK9	Increasing the interaction of PCSK9 and tensin homolog (PTEN), which induced lysosome-mediated PTEN degradation and PI3K/AKT pathway activation.	[127]
zDHHC17	Golgi	Oct4A Smad7	Ensuring Oct4A protein stability; promoting the translocation of Smad7 from the nucleus to the cytoplasm and enhancing its protein stability.	[57, 165]
zDHHC18	Golgi	MDH2 cGAS	Preventing MDH2 ubiquitination and increasing its protein stability; decreasing the interface between cGAS and double-stranded DNA, thereby further obstructing cGAS dimerization.	[166, 167]
zDHHC19	Cytoplasm, Golgi	SQSMT1	Enhancing the affinity of SQSTM1 droplets with the phagophore membrane.	[168]
zDHHC20	Cytoplasm, Golgi, ER	EGFR CD80 AKT	Activating the EGFR signaling; safeguarding the CD80 protein against ubiquitination-mediated degradation, preserving its stability, and ensuring correct localization of CD80 to the PM; activating the PI3K-AKT signaling pathway.	[169–171]
zDHHC21	Golgi, PM	FASN, FYN	Decreasing FASN’s stability and fatty acid synthesis; regulating FYN localization in hair follicles.	[172, 173]
zDHHC22	ER, Golgi	mTOR CCN3	Reducing mTOR stability by palmitoylation and decreasing the activation of the AKT signaling pathway.	[174]
zDHHC23	Golgi	GFAP PHF2	Activating the STAT3 signaling pathway; enhancing ubiquitin-dependent degradation of PHF2.	[130, 175]
zDHHC24	Membrane	—	—	—
APT1	Membrane, Cytoplasm, ER, Nucleus	β-catenin, Scamp1	APT1 and APT2 belong to hydrolysis of FA from S-palmitoylate Cys residues in proteins. APT1 depalmitoylates β-catenin, which modulates renal fibrosis by increasing its abundance and nuclear translocation.	[158, 176]
APT2	Cytoplasm	STAT3, GSDMD	Promotes phosphorylated STAT3 translocation to the nucleus; facilitating GSDMD-NT oligomerization.	[19, 63]
PPT1/2	Lysosome	—	PPT1/2 targets lysosomes through the mannose 6-phosphate receptor-mediated pathway and promotes the degradation of substrate protein by depalmitoylating them within the lysosomes.	[48, 177, 178]
ABHD17A/B/C	Membrane	NLRP3, N-Ras, MAP6	ABHDs are another category of cytosolic depalmitoylases involved in modulating the depalmitoylation of vital proteins on the PM. ABHD17A depalmitoylates NLRP3, inhibiting NLRP3 inflammasome activation.	[100, 179, 180]

### The physiological functions of S-palmitoylation

S-palmitoylation has a significant impact on modulating protein trafficking, membrane localization, stability, signal transduction, and protein conformation [48] (Fig. 1B). The reversible nature of S-palmitoylation is crucial for coordinating protein sorting and facilitating protein transport between organelles [49]. For instance, newly synthesized Ras proteins are subjected to palmitoylation in the Golgi apparatus to enter the secretory pathway, and are then transported to the PM. Once activated in the PM, depalmitoylation occurs, which reduces membrane affinity and enables their transport back to the Golgi apparatus [50]. The dynamic palmitoylation of Ras finely regulates its cycling between the PM and Golgi apparatus, preventing nonspecific retention at the PM and facilitating the transport of activated Ras to the Golgi or ER for downstream signaling [51]. CD36, a key mediator of long-chain fatty acid uptake in multiple cell types, relies on its expression and localization in the PM for proper functioning. S-palmitoylation increases the hydrophobicity of CD36, facilitating its integration into the PM, whereas inhibition of S-palmitoylation results in CD36 accumulation in the ER [21]. DHHC4 and DHHC5 have been identified as mediators of CD36 S-palmitoylation, with DHHC4 promoting CD36 S-palmitoylation in the Golgi and subsequent vesicular trafficking of CD36 to the PM, whereas DHCC5 is responsible for maintaining CD36 in the PM [52]. Furthermore, additional research discovered that CD36 is under the control of dynamic S-palmitoylation during the process of FA uptake [53].

Palmitoylation can decrease the likelihood of substrate proteins being transported to membrane domains containing E3 ubiquitin ligases or decrease the accessibility of lysine residues to these ligases, thereby preventing ubiquitination, inhibiting proteasomal degradation, and enhancing protein stability [54]. For example, S-palmitoylation of PD-L1 at Cys272 residue inhibits its ubiquitination, prevents lysosomal degradation, and increases its binding to PD-1 receptors on T cells, significantly suppressing T-cell cytotoxicity and allowing cancer cells to evade immune surveillance [38]. Palmitoylation protects several proteins, including NOD2 [55], Fas [56], and Oct4 [57], from lysosomal degradation, similar to PD-L1.

Additionally, S-palmitoylation primarily influences the functions of key proteins in various signaling pathways [12, 58]. The related signaling pathways are involved in tumor and immune signaling [59–61]. For example, in the phosphoinositide 3-kinase (PI3K)/protein kinase B (AKT) signaling pathway, AKT, a critical signaling molecule, relies on S-palmitoylation at the Cys344 residue to facilitate its localization to the PM, where it is phosphorylated and activated by 3-phosphoinositide-dependent protein kinase 1 (PDK1) [62]. Pyroptosis is crucial for host defense against pathogens, yet it can also exacerbate inflammatory disorders [63, 64]. Palmitoylation of GSDMD, meditated by DHHC7, promotes its cleavage by caspases and facilitates the trafficking and translocation of GSDMD-NT to the PM for subsequent, followed by depalmitoylation of GSDMD-NT by APT2, which promotes GSDMD-NT oligomerization by unmasking its Cys192 residue [63]. The detailed biological functions of palmitoylation are described in the review by Mesquita [13].

## Roles of s-palmitoylation in digestive system diseases

**Fig. 2:**
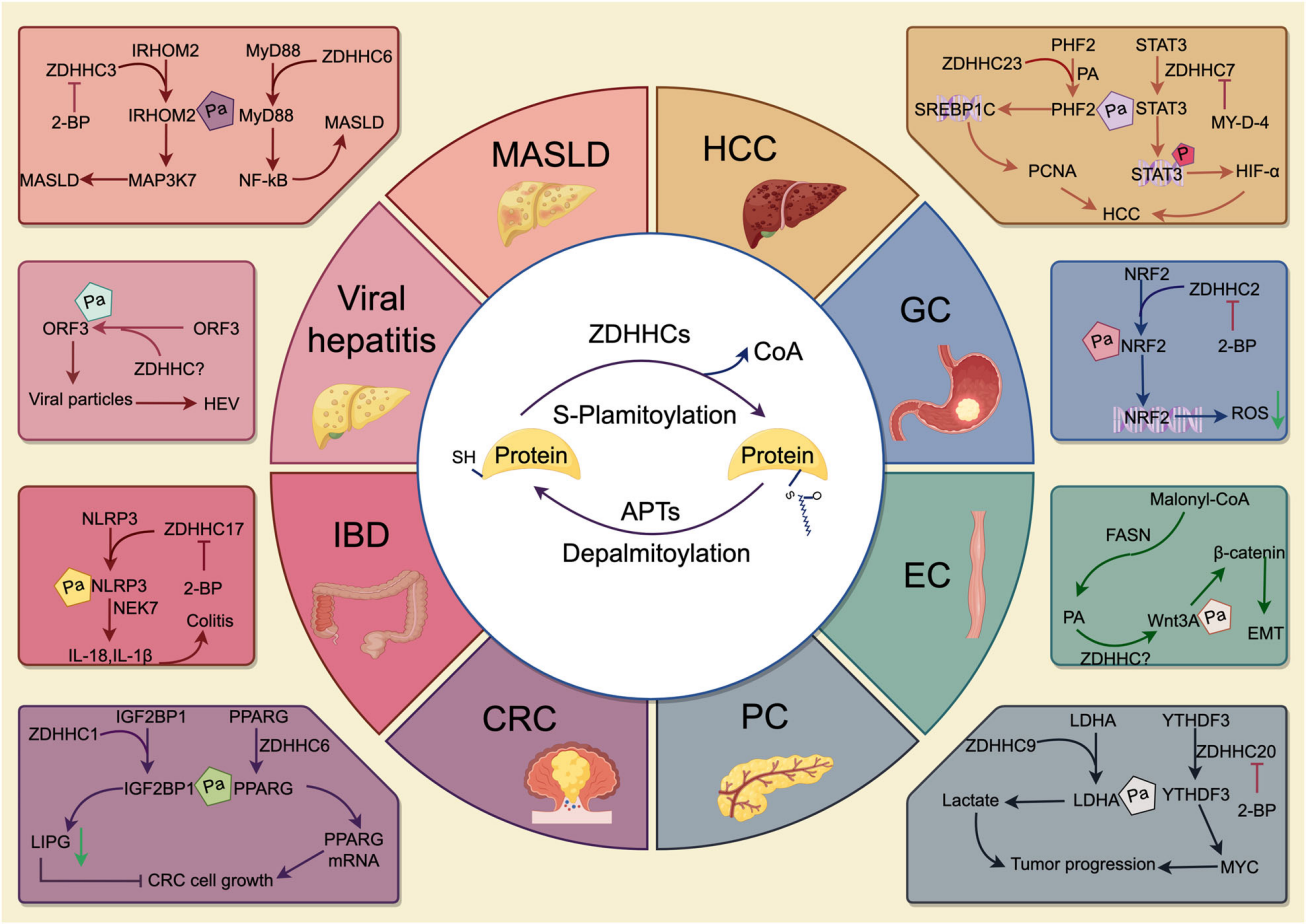
ZDHHCs-mediated S-palmitoylation regulate digestive system diseases. Different ZDHHC enzymes act on different substrates to participate in the occurrence of digestive system diseases, and inhibitors of ZDHHC enzymes (2-BP, MY-D-4) can effectively treat diseases.

Ample reports have emphasized the critical role of palmitoylation in several digestive system diseases, incorporating nonalcoholic steatohepatitis (NASH), IBD, and gastrointestinal cancers (Fig. 2). Palmitoylation influences various pathological processes, such as immune pathways and cancer development. The modulation of specific palmitoylation enzymes, particularly the ZDHHC family, holds potential as a therapeutic strategy for managing diseases of digestive system. The results of these studies are summarized in Table 2.

**Table 2:** Palmitoylation in digestive system diseases.

Disease	Enzyme	Target	Sites	Mechanism	Effects	Refs.
NAFLD	ZDHHC3	IRHOM2	Cys476	ZDHHC3 mediates IRHOM2 palmitoylation at Cys 476, which facilitates its membrane localization and aggregation.	Aggravates fatty acid-induced liver fibrosis and inflammation.	[82]
	ZDHHC5 ZDHHC5	PKCδ RIPK1	N/A Cys257	ZDHHC5 mediates PKCδ palmitoylation promotes its activation. ZDHHC5 mediates RIPK1 palmitoylation at Cys257 and promotes its activation.	Reduces neuroinflammation and improves lipid metabolism. Induces downstream cell death signaling.	[85] [72]
	ZDHHC6	MyD88	Cys113/274	ZDHHC6 mediates MyD88 palmitoylation at Cys113/274; silencing ZDHHC6 contributes to activation of TLR/MYD88 signal pathway by decreasing the palmitoylation of MYD88.	Activates inflammatory response.	[22]
	ZDHHC6	CD36 CD36	Cys3/7/464/466	ZDHHC6 mediates CD36 palmitoylation at Cys3/7/464/466 and promotes its subcellular trafficking.	Enhances fatty acid uptake.	[75]
	ZDHHC7		Cys3/7/464/466	ZDHHC7 mediates CD36 palmitoylation at Cys3/7/464/466 and influences its plasma membrane localization in hepatocytes.	Enhances fatty acid uptake.	[23]
IBD	ZDHHC5	NOD2	Cys495	ZDHHC5 mediates NOD2 palmitoylation at Cys495 and alters its membrane localization.	Activates immune signaling.	[18]
	ZDHHC7	STAT3 STAT3	Cys108	ZDHHC7 mediates STAT3 palmitoylation at Cys108 and promotes it’s the membrane recruitment, phosphorylation and nuclear translocation, thereby enhancing STAT3 activation.	Promotes TH17 cell differentiation and IBD progression.	[19]
	APT2		N/A	APT2 depalmitoylates to the phosphorylated STAT3 and enables it to translocate to the nucleus.	Promotes TH17 cell differentiation.	[19]
	ZDHHC17	NLRP3	Cys419	ZDHHC17 mediates NLRP3 palmitoylation at Cys419 facilitates the interaction between NLRP3 and MIMA-related kinase 7 (NEK7), which promotes NLRP3 activation.	Promotes inflammatory response and IBD development.	[20]
Peritonitis	ZDHHC5	NLRP3 NLRP3 NLRP3 NLRP3	Cys837/838	ZDHHC5 mediated NLRP3 palmitoylation at Cys837/838 enhances NLRP3-NEK7 interaction.	Promotes inflammasome activation and peritonitis.	[100]
	ABHD17A		N/A	ABHD17A depalmitoylates NLRP3.	Alleviates inflammasome activation and peritonitis.	[100]
	ZDHHC7		Cys126	ZDHHC7 mediates NLRP3 palmitoylation at Cys126, which promotes the localization of resting NLRP3 on the trans-Golgi network (TGN) and activates NLRP3 on the dispersed TGN.	Promotes inflammasome activation.	[101]
	ZDHHC12		Cys841	ZDHHC12 mediates NLRP3 palmitoylation at Cys841, promoting NLRP3 degradation via the CMA pathway.	Prevents inflammasome activation.	[87]
GC	ZDHHC2	CKAP4	Cys100	ZDHCC2 mediates CKAP4 palmitoylation at Cys100 promotes its translocation to both the nucleus and the PM.	Antiproliferative response.	[24]
	ZDHHC2	NRF2	Cys514	ZDHHC2 mediates NRF2 palmitoylation at Cys514, which promotes its stabilization and nuclear. translocation.	Activates anti-antioxidant signaling, promoting stomach cancer progression.	[25]
PC	ZDHHC2	CKAP4	Cys100	ZDHHC2 mediates CKAP4 palmitoylation at Cys100, resulting in the localization of CKAP4 inside detergent-resistant membrane (DRM) fractions, activating the PI3K-AKT pathway.	Promotes pancreatic cancer cell proliferation.	[113]
	NA	LRP6	Cys1394/1399	Palmitoylation of LRP6 contributes to the activation of DKK1-CKAP4 signaling by keeping LRP6 localized to lipid rafts.	Promotes pancreatic cancer cell proliferation.	[113]
	ZDHHC5	SSTR5	Cys134	ZDHHC5 mediates SSTR5 tail palmitoylation at Cys134 downregulates its inhibitory effect.	Promotes pancreatic cancer cell proliferation.	[114]
	ZDHHC9	LDHA	Cys163	ZDHHC9 mediated LDHA palmitoylation at Cys163, accelerating LDHA enzyme activity and lactate production.	Increases cell proliferation, tumorigenesis, chemotherapy resistance, and immune escape.	[27]
	ZDHHC20	YTHDF3	Cys474	ZDHHC20 promotes the palmitoylation of YTHDF3 at Cys474 through STAT3, enhancing the stability of oncogenic product MYC mRNA.	Promotes pancreatic cancer progression.	[29]
CRC	ZDHHC1	IGF2BP1	Cys337	ZDHHC1 palmitoylates IGF2BP1 at Cys337 and leads to the downregulation of lipase G (LIPG) expression.	Reduces lipid storage and inhibits colorectal cancer growth.	[32]
	ZDHHC3	PD-L1	Cys272	ZDHHC3 mediates PD-L1 palmitoylation at Cys272 and blocks its lysosomal degradation, which inhibits the interaction between PD-L1 and ESCRT.	Inhibits PD-L1 palmitoylation and enhances T-cell immune responses against tumors.	[38]
	ZDHHC6	PPARγ	Cys313	ZDHHC6 promotes the palmitoylation of PPARγ at Cys313 stabilize and block PPARγ lysosomal degradation, which increasing the expression of ATP citrate lyase (ACLY).	Promotes the synthesis of fresh FA and the formation of tumors.	[30]
	ZDHHC7	Fas	Cys199	ZDHHC7 mediates Fas palmitoylation at Cys199 and enhances its stability on the cell membrane and promotes Fas-dependent apoptotic signaling.	Allows cancer cells to become more resistant to Fas-induced cell death.	[56]
	ZDHHC9	PD-1	Cys192	ZDHHC9 mediates PD-1 palmitoylation at Cys192 to stabilize its protein level and promotes the interaction between PD-1 and Rab11.	Facilitates tumor growth.	[122]
HCC	ZDHHC6	AEG-1	Cys75	ZDHHC6 mediates AEG-1 palmitoylation at Cys75 to promote the interaction AEG-1 and the E3 ubiquitin ligase FBXW7, which enhances the ubiquitination level and proteasome-mediated degradation of AEG-1.	Suppresses HCC progression.	[33]
	ZDHHC16	PCSK9	Cys600	ZDHHC16 mediates PCSK9 palmitoylation at Cys600 to dramatically increase the interaction PCSK9 and tensin homolog (PTEN), which induces lysosome-mediated PTEN degradation and PI3K/AKT pathway activation.	Induces sorafenib resistance in liver cancer.	[127]
	ZDHHC17/24	AKT	Cys77/224	ZDHHC17/24 mediated AKT palmitoylation at Cys77/224 to prevent ATK from assembling into an inactive polymer, which alters AKT on the cell membrane.	Antagonizes liver tumorigenesis.	[34]
	ZDHHC23	PHF2	Cys23	ZDHHC23 mediates PHF2 palmitoylation at Cys23, augmenting its ubiquitination, which contributes to stable expression of SREBP1c and raise FFAs in HCC cells.	Leads to poor HCC prognosis.	[130]
	ZDHHC7	STAT3	Cys108	ZDHHC7 mediates STAT3 palmitoylation at Cys108, increasing the transcriptional activity of STAT3, which contributes to increasing the expression of HIF1α.	Promotes the malignancy of HCC in mice.	[35]
EC	ZDHHC?	Wnt3A	Cys77	Promotes Wnt3A membrane localization and the translocation of β-catenin into the nucleus.	Activates Wnt3A/β-catenin axis and induces EMT phenotyp.e	[138]
Viral hepatitis	ZDHHC?	ORF3	Cys18/21	Release ORF3 vesicles from hepatocytes and promotes ANXA2 binding to palmitoylated ORF3.	Directs hepatitis E virus ORF3 sorting into vesicles and virion production.	[140]

This table illustrates the palmitoylases and depalmitoylases in digestive system diseases (e.g., metabolic dysfunction-associated steatotic liver disease, inflammatory bowel diseases, and colorectal cancer) and their mechanisms.

### Metabolic dysfunction associated steatotic liver disease (MASLD)

Nonalcoholic fatty liver disease (NAFLD) is a chronic progressive liver disease characterized by excessive lipid accumulation, inflammation, and hepatocyte injury, primarily driven by disturbances in glucose and lipid metabolism. In late 2023, three large pan-nation liver associations proposed the term ‘metabolic dysfunction-associated steatotic liver disease’ (MASLD) to replace NAFLD [65]. MASLD has a global prevalence of 38.2% and poses a major threat to human health [66–68]. The development and progression of MASLD is critically linked to an imbalance in hepatic FA metabolism. The abnormal activation of FA synthesis pathways results in excessive hepatic fat accumulation. On the other hand, insulin resistance (IR) promotes lipolysis, which exacerbates the accumulation of free FAs in the liver. The resulting lipotoxicity from excessive free FA and its metabolites induces hepatic inflammation, advancing the disease to NASH [69]. Cell death and inflammation are key pathological outcomes in MASLD [70, 71]. Recent studies have revealed that some key molecules complicated with lipid metabolism, inflammatory signaling and cell death are modulated by palmitoylation, which is pivotal in the pathogenesis of MASLD [21, 22, 72].

The function of CD36 is thoroughly linked to its subcellular localization, which can be influenced by palmitoylation [21, 73]. Recent research has indicated that the absence of CD36 palmitoylation promotes its mitochondrial distribution and enhances its interaction with long chain-acyl-coenzyme A synthetase 1 (ACSL1). The binding of CD36 to ACSL1 acts as a “bridge” between long-chain fatty acids (LCFAs) and mitochondria, facilitating the transport of LCFA to ACSL1 and thereby enhancing fatty acid oxidation (FAO) in hepatocytes and mitigating lipid accumulation in NAFLD [74]. ZDHHC6 and ZDHHC7 are key enzymes that catalyze the palmitoylation of CD36 in hepatocytes [23, 75]. Kruppel-like factor 10 (KLF10), which is upregulated in NASH livers, has been shown to increases the transcriptional level of ZDHHC7, thereby promoting CD36 palmitoylation and exacerbating hepatic lipid accumulation and inflammation [23]. Another study revealed that in NASH mouse livers, upregulation of selenoprotein K (SelK) enhances the catalytic efficiency of ZDHHC6 through its interaction with the src homology 3 (SH3) domains. Moreover, it accelerates the integration of palmitoylated CD36 into cytoplasmic coat protein complex II (COPII) vesicles, promoting the transport of CD36 from the ER to the Golgi apparatus and thus accelerating disease progression [75].

The Toll-like receptor 4 (TLR4)-myeloid differentiation primary response 88 (MyD88)-nuclear factor κB (NF-κB) pathway is stimulated by excessive levels of saturated fatty acids (SFA) in NAFLD, leading to increased expression of downstream inflammatory cytokines such as tumor necrosis factor (TNF)-α, interleukin (IL)-6, and IL-1β and thereby contributing to the pathogenesis of NAFLD [76]. MyD88 activation requires palmitoylation at Cys113 and Cys274, which is catalyzed by ZDHHC6 [22]. Endogenously synthesized FAs mediated by FASN and exogenous FAs taken up by hepatocytes via CD36 synergistically promote MyD88 palmitoylation [22]. FASN also regulates membrane cholesterol levels to facilitate the influx of exogenous FAs and maintain intracellular PA levels [22]. In an animal study of NASH, inhibition of the FASN pathway has been found to attenuate MyD88-dependent TLR signaling and subsequent inflammatory responses [22, 77].

Inactive rhomboid protein 2 (IRHOM2), a non-functional member of the rhomboid protease family, was linked to the progression of NAFLD/NASH [78, 79]. Further research has highlighted the role of IRHOM2 ubiquitination in regulating NASH progression, suggesting that targeting IRHOM2 could offer therapeutic potential in the management of NAFLD/NASH treatment [80, 81]. Recent study of liver tissue samples from patients with NASH demonstrated a positive correlation between high levels of ZDHHC3 protein and the severity of the NASH phenotype [82]. Mechanistic investigations showed that ZDHHC3 palmitoylates IRHOM2 at Cys476, facilitating its membrane localization and aggregation. Interestingly, elevated circulating free FA levels induced by a high-fat diet showed to directly enhance IRHOM2 palmitoylation, thereby promoting its transport. Conversely, the competitive PAT inhibitor 2-bromopalmitate (2-BP) significantly reduces palmitoylated IRHOM2 accumulation, thereby alleviating fatty acid-induced liver fibrosis and inflammation [82].

In addition to proteins associated with lipid metabolism, the neuroendocrine system is essential for regulation of lipid metabolism. The hypothalamus is an essential brain region that links the neuroendocrine system to peripheral physiological activities [83]. Previous studies have shown that PKCδ is activated by lipid metabolites and is associated with lipid-induced liver diseases [84]. Recent findings indicate that palmitoylation of PKCδ by ZDHHC5 in hypothalamic microglia regulates peripheral lipid metabolism via the hypothalamus-liver axis. Artemether (an antimalarial drug) inhibits PKCδ palmitoylation by blocking its interaction with ZDHHC5, thereby reducing neuroinflammation and improving lipid metabolism. This study presents a novel therapeutic strategy for fatty liver disease [85].

**Fig. 3:**
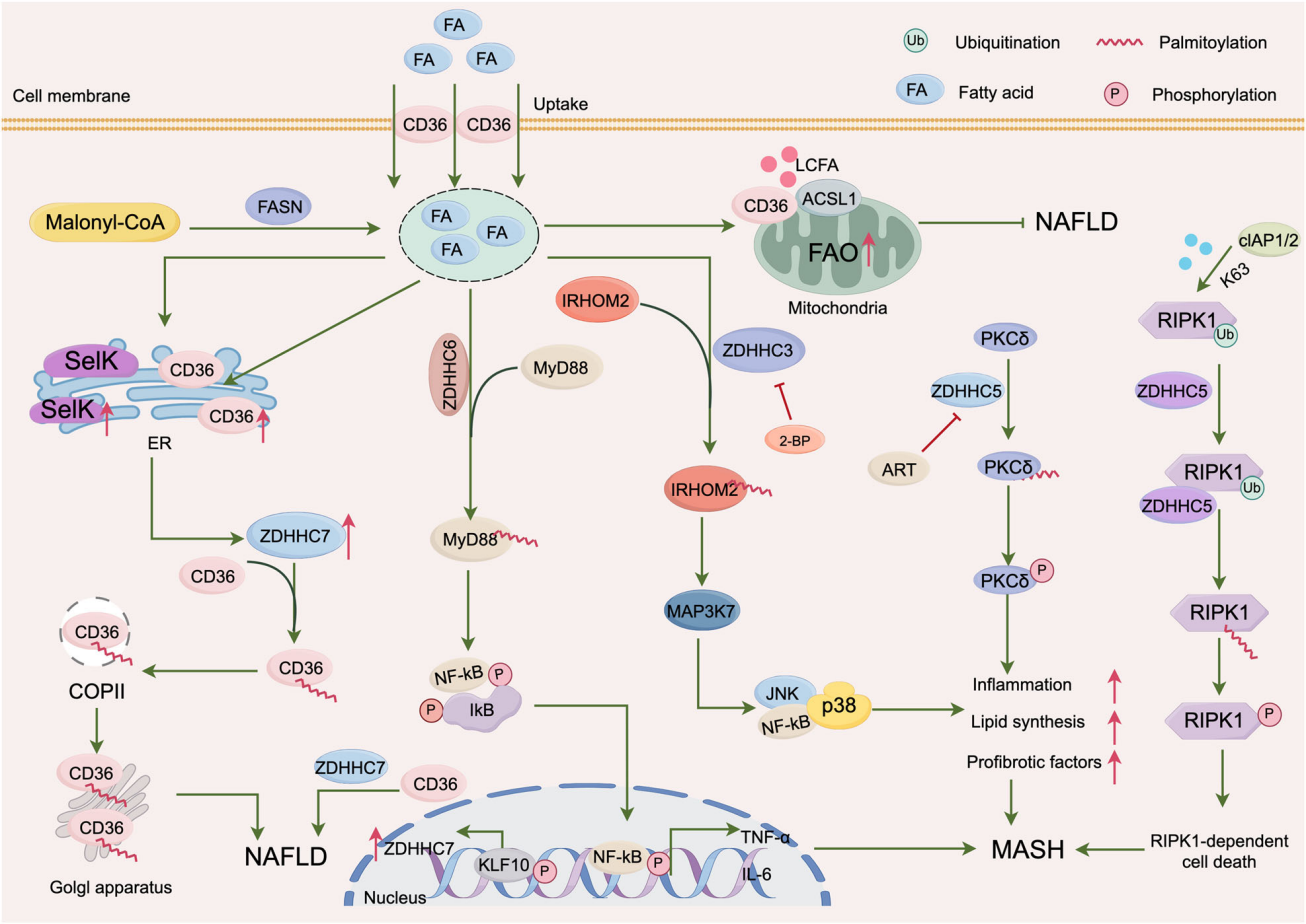
ZDHHCs-mediated S-palmitoylation regulate MASLD progression. Fatty acids enter hepatocytes through the uptake of CD36 and play a role in different ZDHHC enzymes to reduce or aggravate the occurrence of MASLD. In addition, it participates in the progress of MASLD by activating inflammatory pathways and cell death under the action of different ZDHHC enzymes.

Furthermore, a recent study demonstrated that ZDHHC5-mediated palmitoylation of RIPK1 facilitated cell death, resulting in hepatic inflammation and liver damage in MASH [72]. Autophagy is implicated in digestive system diseases, and palmitoylation is important for regulating autophagy [86, 87]. Autophagy-related protein 16-like 1 (ATG16L1) is indispensable for the process of autophagy and autophagosome formation. Recent study indicated that ZDHHC7-mediated palmitoylation of ATG16L1 promoted LC3 lipidation and autophagosome formation [88]. Interestingly, a study shown that macrophage ATG16L1 expression suppresses MASH progression by promoting lipophagy [89]. Further study suggested that ATG16L1 knockout enhanced stimulator of interferon genes (STING) palmitoylation, thus promoted STING trafficking from the endoplasmic reticulum to the Golgi, and activated downstream STING signaling, promoting proinflammatory and profibrotic cytokines secretion, resulting in hepatic steatosis and hepatic stellate cells activation [89]. Figure 3. summarizes these regulatory mechanisms.

### IBD

IBD is a chronic persistent inflammatory disorder that primarily affects the gastrointestinal system, encompassing both Crohn’s disease (CD) and ulcerative colitis (UC). Dysregulation of the intestinal immune system is considered a critical mechanism underlying the pathogenesis of IBD. Aberrant immune responses, particularly overactivation of T cells and macrophages, result in the production of pro-inflammatory cytokines such as IL-6 and TNF-α, which exacerbate intestinal inflammation and compromise the integrity of the intestinal barrier [90–92].

#### CD

NOD2, an intracellular pattern recognition receptor, is a key susceptibility gene for CD. Under conditions of immune balance, NOD2 facilitates the production of antimicrobial peptides (e.g., α-defensins) via Paneth cells and mucus secretion by goblet cells, thus maintaining the integrity of the intestinal epithelial barrier [93]. When intestinal bacteria invade epithelial cells, NOD2 recognizes microbial-associated muramyl dipeptide (MDP) and is recruited to the membrane of bacteria-containing endosomes, where it activates the NF-κB pathway, leading to the production of inflammatory cytokines (e.g., IL-1β) that help defend against bacterial invasion. Concurrently, NOD2 prevents the overactivation of the TLR2/NF-κB signaling pathway, which would otherwise result in excessive inflammation [94]. The precise membrane targeting of NOD2 depends on the palmitoylation of multiple cysteine residues by ZDHHC5 [18]. Common loss-of-function mutations in NOD2 (e.g.,3020insC, R702W, L248R, A612T, A755V, and R1019stop) result in a 70%–90% reduction in S-palmitoylation, impairing the endosomal membrane targeting of NOD2. Consequently, these NOD2 mutants fail to inhibit the overactivation of the TLR2/NF-κB signaling pathway and its downstream inflammatory mediators, including IL-12, IL-1β, and interferon- (IFN)-γ, leading to further damage to the intestinal epithelial cells. This mode of dysregulation significantly increases the risk of developing CD [18].

Additionally, ZDHHC5 was recently shown to mediate the palmitoylation of NOD2, inhibiting its interaction with the selective autophagy cargo receptor SQSTM1/p62. This inhibition prevents NOD2 degradation via selective autophagy, thereby enhancing the protein stability and promoting NOD2-mediated downstream inflammatory responses [55]. However, when the R444C mutation was introduced into NOD2, the mutant (NOD2s-R444C) exhibited increased binding to ZDHHC5, thereby exacerbating the inflammatory response. Inhibiting the palmitoylation of NOD2s-R444C can mitigate the excessive inflammatory response caused by this mutant. Interestingly, the R471C/R444C mutation in NOD2 is more prevalent in Asian populations, and has been frequently identified in patients with autoimmune inflammatory diseases, including IBD. These findings could be instrumental in improving the diagnosis and precision of treatment of patients with NOD2 mutation-related autoimmune inflammatory diseases.

STAT3-mediated differentiation of TH17 cells is crucial for the pathogenesis of IBD [19]. STAT3 is a transcription factor that, upon phosphorylation by JAK2 in response to extracellular IL-6 stimulation, translocate to the nucleus to activate the transcription of downstream genes such as RORC and IL-17, thereby promoting TH17 cell differentiation. Research has indicated that ZDHHC7 facilitates the palmitoylation of STAT3 at Cys108, facilitating its translocation to the cell membrane, where it is more readily phosphorylated by JAK2. However, entry of phosphorylated STAT3 into the nucleus requires depalmitoylation [19]. Further studies have identified APT2 as the depalmitoylation enzyme for STAT3, with phosphorylation at Y705 being a prerequisite for STAT3 depalmitoylation. This ensures the unidirectional movement of the palmitoylation-depalmitoylation cycle, thereby facilitating STAT3 activation. In summary, these studies provide compelling evidence that targeting palmitoylation of key molecules, including STAT3 and NOD1/2, may serve as promising therapeutic strategy for the CD.

#### UC

Mutations in NLRP3 leading to inflammasome hyperactivation are significant risk factors for early-onset IBD [95]. Recent studies have revealed that ZDHHC17 promotes NLRP3 activation by palmitoylating the Cys419 residue, thereby facilitating the interaction between NLRP3 and NEK7, which drives the IBD progression. In an animal study of colitis, the use of 2-bromopalmitate (2-BP), a palmitoylation inhibitor, improved survival rates and reduced weight loss, indicating that regulating NLRP3 palmitoylation is a prospective therapeutic strategy for NLRP3-driven inflammatory diseases [20]. Moreover, studies on 2’-fucosyllactose (2’-FL) have demonstrated its potential to mitigate UC by restoring the integrity of the intestinal mucosal barrier and suppressing inflammation through the inhibition of STAT3 palmitoylation and phosphorylation. This inhibition is crucial, since it prevents the degradation and activation of STAT3, thereby reducing inflammation and highlighting the significance of STAT3 in mediating the therapeutic effects of 2’-FL in UC [96]. In parallel, Jiang et al. identified NU6300 as a novel inhibitor of GSDMD that exerts its effects by covalently binding to cysteine-191, thereby blocking GSDMD cleavage and its subsequent palmitoylation and membrane localization. This mechanism not only disrupts the role of GSDMD in pyroptosis but also selectively interferes with upstream inflammasome signaling. These results highlight the therapeutic potential of NU6300 in treating inflammatory conditions such as colitis, offering a new avenue for intervention [97]. These findings collectively advance our understanding of the molecular mechanisms underpinning inflammatory bowel diseases and propose novel therapeutic strategies centered on the inhibition of palmitoylation of key inflammatory pathways.

### Peritonitis

NLRP3 is a crucial cytoplasmic pattern recognition receptor that responds to various pathogen-associated molecules and danger signals, and plays a major role in numerous diseases [98, 99]. Palmitoylation mediates the inflammasome functions of NLRP3. Study showed that ZDHHC5 mediates the palmitoylation of NLRP3 at Cys837/838, which enhances the assembly and activation of the NLRP3 inflammasome, while ABHD17A removes this palmitoylation [100]. Their findings indicate that restoring the palmitoylation balance of NLRP3 could be a prospective therapeutic target for diseases driven by NLRP3 activation. ZDHHC7-mediated palmitoylation of NLRP3 at Cys126 is essential for the activation of the NLRP3 in macrophages. Studies have suggested that the loss of ZDHHC7 protects against peritonitis induced by monosodium urate (MSU) crystals. These findings demonstrate that targeting ZDHHC7-mediated palmitoylation of NLRP3 at Cys126 may effectively mitigate NLRP3 inflammasome-mediated inflammation in mice, presenting a promising therapeutic approach for inflammasome-associated human diseases [101]. Additionally, studies have identified that ZDHHC12 mediates the palmitoylation of NLRP3 at Cys841, facilitating its degradation via the chaperone-mediated autophagy pathway and thereby preventing excessive inflammasome activation [87]. A deficiency in ZDHHC12 can activate the NLRP3 and increase IL-1β release in mice, exacerbating Alum-induced peritonitis and lipopolysaccharide-induced sepsis.

### Digestive cancers

Recent advances have emphasized the close association between palmitoylation and various types of cancers [102–104]. Numerous key oncogenic proteins, including epidermal growth factor receptors (EGFR), Ras family GTPases, AKT, Wnt proteins, astrocyte elevated gene (AEG)-1, and PD-L1, undergo palmitoylation. These substrate proteins are involved in multiple aspects of tumorigenesis, including cell proliferation and survival, migration and invasion, signal transduction, and tumor immunity [14, 59, 105]. In this section, we review the relationship between palmitoylation and cancers of the stomach, colorectum, liver, and pancreas, emphasizing the potential of palmitoylation for treatment of gastrointestinal tumors.

#### Gastric cancer

Gastric cancer (GC) is a malignancy characterized by rapid progression, poor response to treatment strategies, and high mortality rates [106, 107]. However, research on protein palmitoylation in the context of GC is still in its early stages. Studies have analyzed differences in the expression of ZDHHC2 in GC tissues and adjacent normal tissues, revealing that ZDHHC2 expression is downregulated in gastric adenocarcinoma. Notably, significant differences were observed in lymph node metastasis and histological grading between the high and low ZDHHC2 expression groups [108], which is consistent with the findings in liver cancer [109]. Further survival analysis of 472 patients with GC revealed statistically significant differences in the 5-year survival rates between the low and high ZDHHC2 expression groups (2.21% and 2.26%, respectively). These dates suggested that ZDHHC2 may serve as a prospective prognostic biomarker for GC [108]. Cytoskeleton-associated protein 4 (CKAP4) is a type II transmembrane protein located in the ER [110]. Research has demonstrated that ZDHHC2 regulates antiproliferative signaling by mediating the palmitoylation of CKAP4, indicating that ZDHHC2 may function as a tumor suppressor [24].

Recent evidence indicates that ZDHHC2 promotes GC growth and reduces reactive oxygen species (ROS) levels by stabilizing and translocating NRF2 into the nucleus through palmitoylation of NRF2 at the Cys514 site, which prevents its ubiquitination and subsequent degradation by the proteasome [25]. Additionally, comparisons of ZDHHC14 mRNA expression between sclerosing GC tissues and normal tissues revealed that ZDHHC14 was expressed in GC tissues, but not in normal tissues. Functional experiments further indicated that knocking down ZDHHC14 significantly inhibited GC invasiveness, whereas overexpression of ZDHHC14 enhanced cell adhesion and rapid migration [26]. The expression of ZDHHC14 is related to the regulation of integrin α5 and β1 subunit mRNA and protein, with high ZDHHC14 expression primarily promoting tumor cell migration and invasion.

#### Pancreatic cancer

Previous studies have confirmed that the overexpression of CKAP4 or low-density lipoprotein receptor-related protein 6 (LRP6) promotes the progression of pancreatic cancer [111, 112]. Interestingly, CKAP4 was palmitoylated at Cys100 by ZDHHC2, whereas LRP6 was palmitoylated at Cys1394 and Cys1399. Mechanistically, palmitoylation induces the localization of CKAP4 and LRP6 within detergent-resistant membrane (DRM) domains, subsequently activating the PI3K-AKT pathway and promoting cell proliferation [113]. Somatostatin receptor subtype 5 (SSTR5) is an antiproliferative receptor found in pancreatic cells. ZDHHC5-mediated palmitoylation of SSTR5 at Cys134 inhibits its antiproliferative effects. Lomitapide, an inhibitor of ZDHHC5, reduces the growth and proliferation of pancreatic cancer cells both in vitro and in mouse models [114].

Protein palmitoylation has also been implicated in the immunosuppressive tumor microenvironment and in antitumor immunotherapy. For example, ZDHHC3-mediated palmitoylation of PD-L1 prevents its ubiquitin-mediated degradation, leading to increased PD-L1 expression on the cell surface [38]. Additionally, ZDHHC9 has been identified as having a suppressive effect on antitumor immunity [115]. ZDHHC9 is associated with inhibition of the pancreatic tumor microenvironment along with the K-Ras gene [115]. In ZDHHC9-deficient mouse models, pancreatic tumors exhibit a higher proportion of activated effector T cells, shifting the tumor microenvironment from a suppressive to a pro-inflammatory state. Metabolic reprogramming is another hallmark of tumorigenesis [116]. Another study demonstrated that ZDHHC9-mediated palmitoylation of lactate dehydrogenase A (LDHA) at Cys163 enhanced LDHA enzyme activity, thereby expediting glycolysis and lactate generation, which subsequently promoted cell proliferation and tumorigenesis [27].

**Fig. 4:**
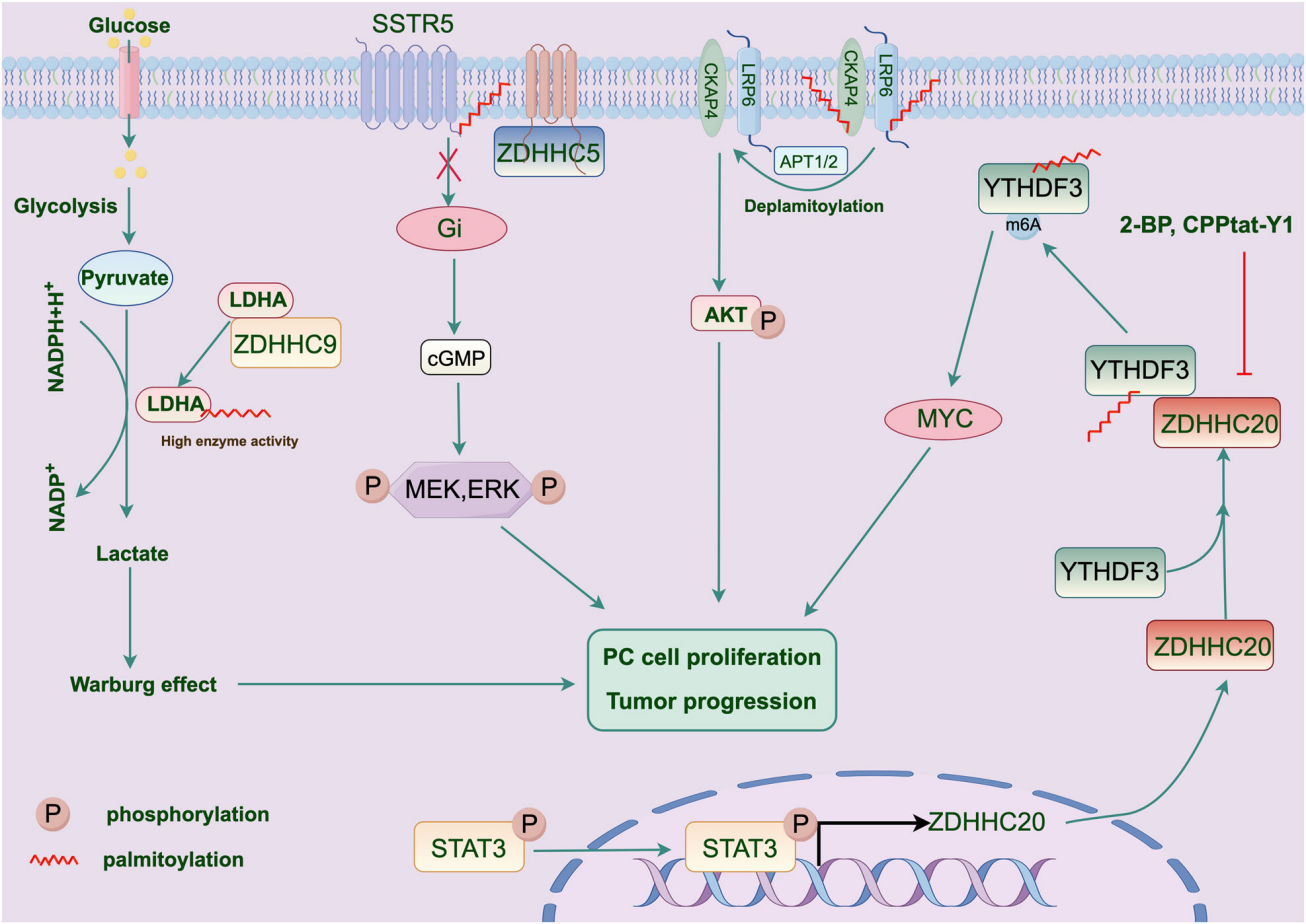
ZDHHCs-mediated S-palmitoylation regulate pancreatic cancer progression. Multiple key oncogenic proteins (MYC, ERK and AKT) and pathways (lactate metabolism) were regulated by palmitoylation, thereby influence pancreatic cancer progression.

Recent research has suggested that ZDHHC20 is important for the interaction between cancer cells and the immune system [28]. Through shRNA screening, ZDHHC20 was identified as being essential for pancreatic cancer metastasis. This was further validated in ZDHHC20 knockout mouse models, where tumor cells could form tumors in immunodeficient animals (primarily due to natural killer [NK] cell deficiency), but not in immunocompetent mice. These findings suggest a potential therapeutic approach for preventing the spread of pancreatic cancer. Similarly, another study revealed that ZDHHC20 is abnormally increased in pancreatic cancer tissues and is related to poor prognosis. Mechanistically, ZDHHC20 facilitates the palmitoylation of YTHDF3 at Cys474 via STAT3, which enhances the stability of MYC mRNA, thereby driving the proliferation and invasion of pancreatic cancer [29]. Figure 4. describes a potential mechanism.

#### Colorectal cancer

PA, the principal substrate for palmitoylation, has a critical role in the progression of colorectal cancer (CRC) through regulation of its level [117]. Acyl-CoA oxidase 1 (ACOX1) suppresses CRC progression by modulating PA reprogramming. Zhang et al. discovered that DUSP14 dephosphorylates ACOX1 at serine 26, enhancing ACOX1 polyubiquitination and subsequent proteasomal degradation, resulting in an increase in the substrate PA [31]. The accumulating PA enhances β-catenin palmitoylation at Cys466, thereby inhibiting CK1- and Gsk3-mediated β-catenin phosphorylation and subsequent β-Trcp-mediated proteasomal degradation. Therefore, targeting PA metabolism and β-catenin palmitoylation may represent novel therapeutic strategies for treating CRC [31]. Furthermore, Zhang et al. observed that ZDHHC1 expression is downregulated in CRC tissues and that low ZDHHC1 levels are correlated with a poor prognosis. Mechanistically, ZDHHC1 palmitoylates IGF2BP1 at Cys337, leading to the downregulation of lipase G (LIPG) expression and thus inhibiting CRC growth [32]. Similarly, Shan et al. demonstrated that ZDHHC6 directly palmitoylates and stabilizes peroxisome proliferator-activated receptor-gamma (PPARγ) at the Cys313 site within its DNA-binding domain, enhancing its nuclear translocation and activating the ATP citrate lyase (ACLY) transcription-related metabolic pathway, and thereby promoting fatty acid biosynthesis and CRC progression. Inhibition of ZDHHC6 reduces cancerous effects, underscoring its potential as a promising target for CRC by disrupting lipid synthesis [30].

Emerging data have implicated palmitoylation in apoptosis, which contributes to the progression of CRC [118]. For instance, Fas (CD95), an essential death receptor involved in apoptosis, undergoes palmitoylation at Cys199 by ZDHHC7, enhancing its stability on the cell membrane and promoting Fas-dependent apoptotic signaling [56]. Additionally, palmitoylation of estrogen receptors can influence their interactions with signaling proteins, thereby affecting estrogen receptor-regulated apoptotic pathways in CRC cells. A study on the colorectal adenocarcinoma DLD-1 cell line indicated that palmitoylation of estrogen receptor β targets it to the PM and promotes its interaction with caveolin-1, which is necessary for estradiol-mediated activation of the pro-apoptotic p38 signaling cascade [119]. The rapid and sustained activation of p38/MAPK by estradiol is crucial for upregulating estrogen receptor β levels in DLD-1 CRC cells [120].

**Fig. 5:**
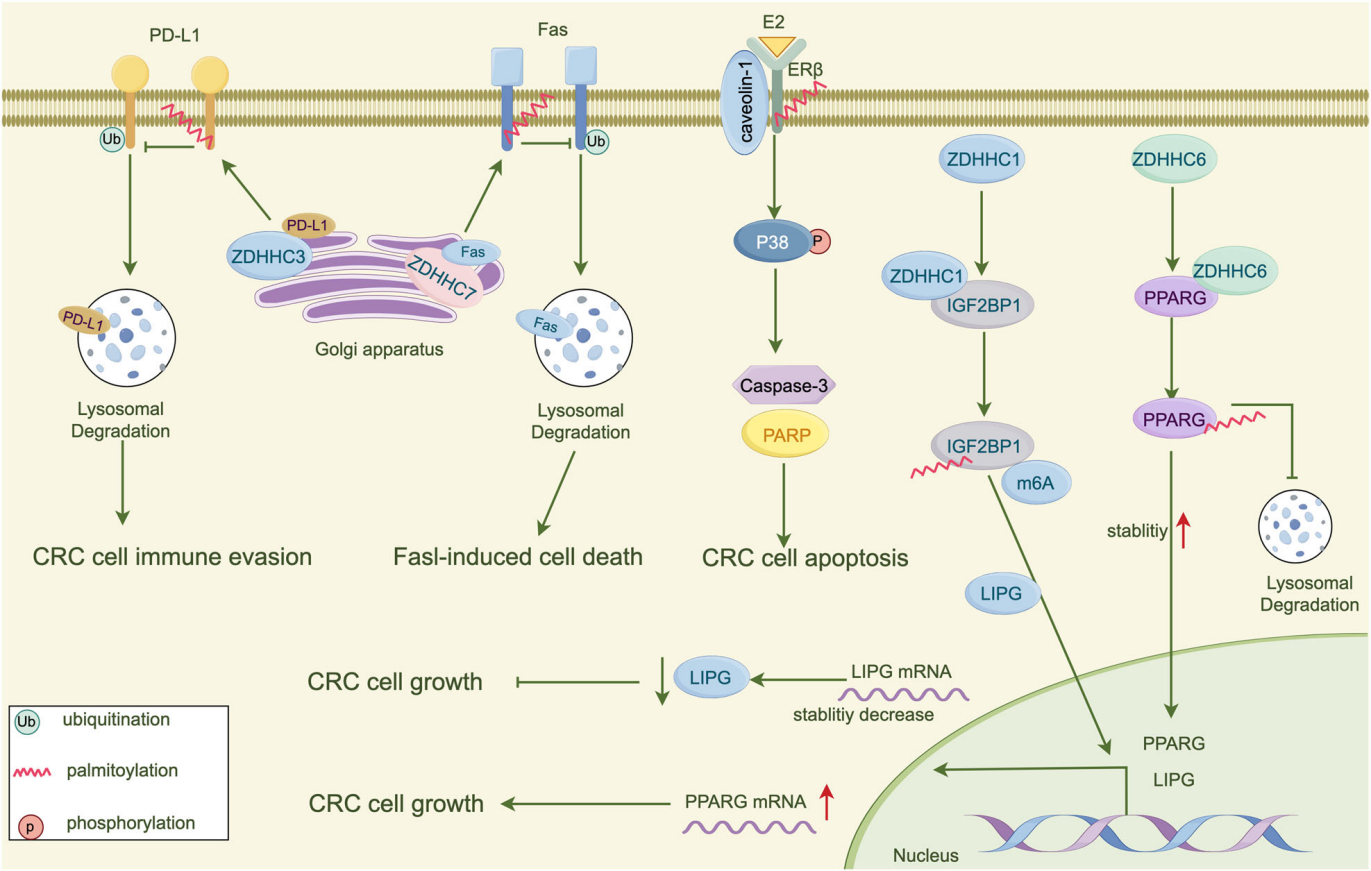
ZDHHCs-mediated S-palmitoylation regulate CRC progression. Palmitoylation blocks lysosomal degradation of PD-L1, Fas, PPARG, enhancing their stability, thereby modulating CRC cell immune evasion, apoptosis, proliferation.

PD-1, a transmembrane glycoprotein encoded by PDCD1, interacts with PD-L1 to inhibit T-cell function. Typically, the PD-1/PD-L1 pathway is important for keeping immune tolerance. However, in tumor immunity, the interaction between PD-1 and PD-L1 is the primary mechanism by which tumor cells evade immune surveillance [121]. Multiple findings have demonstrated that palmitoylation is crucial for stabilizing PD-1/PD-L1 surface expression. For example, experiments in CRC cell lines revealed that PD-1 palmitoylation is mediated by ZDHHC9 at Cys192 [122]. Similarly, studies on breast cancer cells found that ZDHHC9 mediates PD-L1 palmitoylation at Cys272 within the cytoplasmic domain [123]. However, opinions regarding PD-L1 palmitoylation vary, with some studies suggesting that ZDHHC3 is responsible for this modification by targeting the Cys272 site in the PD-L1 cytoplasmic domain [38]. The role of PD-L1 palmitoylation is complex and variable, and its specific function may depend on the tumor type and palmitoylation site. Figure 5 describes a potential mechanism.

#### Hepatocellular carcinoma

CD44, a multifaceted transmembrane adhesion glycoprotein, closely associated with epithelial-mesenchymal transition (EMT), significantly influencing tumor initiation, progression, and metastasis [124]. Recent findings showed that elevated cholesterol levels promote the lipid raft localization of CD44 in a palmitoylation-dependent manner, disrupting CD44-Ezrin interactions, and ultimately decreasing the migration and metastasis of hepatocellular carcinoma (HCC) cells [125]. AEG-1, which is overexpressed in many cancers, is involved in activation of multiple pro-survival and proliferative pathways, including MAPK/ERK, PI3K/AKT, Wnt/β-catenin, and NF-κB [126]. Palmitoylation of AEG-1 at the Cys75 residue is dynamically regulated by ZDHHC6 and PPT1/2 and is crucial for modulating HCC cell proliferation. In vivo study of HCC, the deficiency of AEG-1 palmitoylation was shown to accelerates diethyl-nitrosamine (DEN)-induced HCC progression [33]. The lack of palmitoylation may inhibit the ubiquitination-mediated degradation of AEG-1 and promote RNA-induced silencing complex activity through interactions with staphylococcal nuclease domain-containing protein 1. Hydroxychloroquine, a PPT1 inhibitor, has been shown to target PPT1 and increases AEG-1 palmitoylation levels, thereby inhibiting the growth of HCC cells [33].

In sorafenib-resistant HCC, abnormal expression of proprotein convertase subtilisin/kexin type 9 (PCSK9) promotes chemoresistance by stimulating AKT-S473 phosphorylation. ZDHHC16-mediated palmitoylation of PCSK9 facilitates its interaction with PTEN, thereby increasing lysosome-mediated PTEN degradation and the subsequent activation of AKT. The inhibiting of PCSK9 palmitoylation may play a crucial role in overcoming sorafenib resistance [127]. A novel PCSK9-derived peptide that competitively inhibits PCSK9 palmitoylation was shown to reduce AKT phosphorylation and improvise sorafenib resistance in HCC. Additionally, targeting PCSK9 is associated with ferroptosis, further enhancing its role in HCC treatment [128]. The long non-coding RNA DUXAP8 has shown to enhance the palmitoylation of SLC7A11 at the Cys414 residue, preventing its lysosomal degradation, thereby boosting its function and inhibiting ferroptosis [129].

PA facilitates AKT activation through palmitoylation, with ZDHHC17/24 mediating the palmitoylation of AKT at the Cys224 site and thereby promoting HCC progression [34]. Thus, limiting PA synthesis and targeting ZDHHC17/24 may be an effective strategy for HCC treatment. ZDHHC23-mediated palmitoylation of plant homeodomain finger protein 2 (PHF2) enhances its ubiquitin-dependent degradation, directly destabilizing sterol regulatory element-binding protein 1c (SREBP1c) and decreasing SREBP1c-dependent FA synthesis. Interestingly, SREBP1c promotes the entry of FFAs into HCC cells, with subsequent PA-induced activation of the PHF2/SREBP1c axis [130]. Therefore, monitoring the PA levels in the diet of patients with HCC is essential.

ZDHHC2 overexpression has been shown to inhibit the proliferation, migration, and invasion of HCC cells in vitro, highlighting its role as a tumor suppressor in HCC metastasis and recurrence [109]. Consistent with this finding, elevated ZDHHC7 expression was associated with poor clinical staging and prognosis. Further mechanistic studies revealed that ZDHHC7 catalyzes STAT3 palmitoylation, driving its migration to the cell membrane where it is activated. STAT3 activation leads to the transcription of HIFA, which is also a transcription factor for ZDHHC7, thus forming a positive feedback loop that facilitates HCC progression [35]. The ZDHHC7 inhibitor S-(2-acetamidoethyl) 2-bromohexadecanethioate (MY-D-4) has been shown to effectively suppress HCC growth, suggesting that ZDHHC7 could be a novel molecular marker of HCC. Furthermore, the inhibition of FASN has been found to upregulate MHC-I levels in HCC by decreasing its palmitoylation and subsequent lysosomal degradation. This upregulation enhances antigen presentation and CD8 ^+^ T-cell cytotoxicity, offering a potential synergistic effect when combined with immune checkpoint inhibitors [131]. These findings suggest that integrating FASN inhibitors with PD-L1 blockade represents a promising strategy for improving the efficacy of immunotherapy in HCC.

**Fig. 6:**
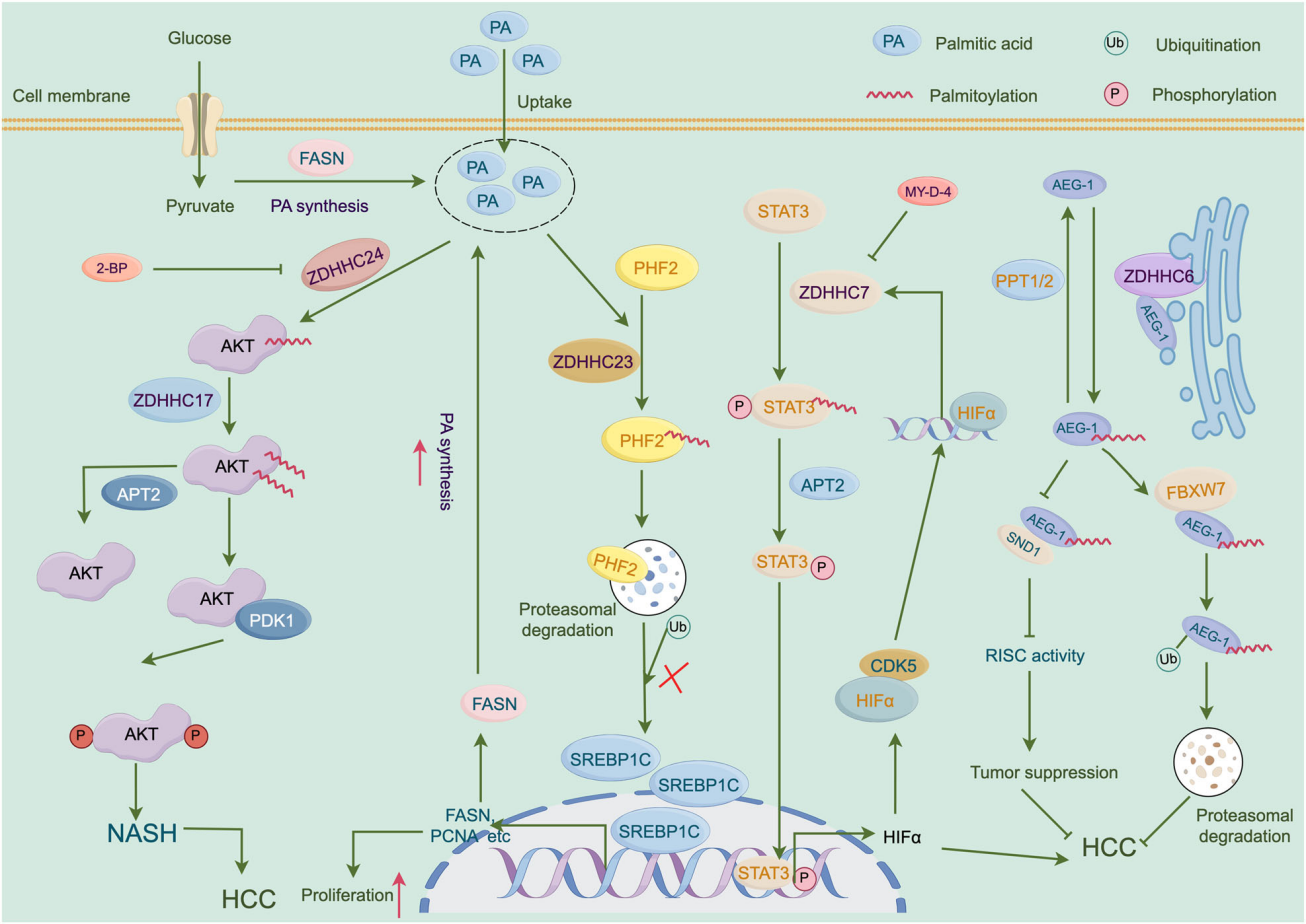
ZDHHCs-mediated S-palmitoylation regulate HCC progression. PA is very important for palmitoylation. When PA levels increased, ZDHHC24 mediating the palmitoylation of AKT and ZDHHC23 promoting PHF2 palmitoylation, and thereby promoting HCC progression. In addition, ZDHHC7 mediates STAT3 palmitoylation, increasing the transcriptional activity of STAT3, which contributes to increasing the expression of HIF1α; the expression of HIF1α positively regulate ZDHHC7.

Previous dates have demonstrated that PPT1 is upregulated in multiple cancers, including HCC, and links to a poor prognosis [132–134]. Recently, a small orally bioavailable molecule, GNS561/Ezurpimtrostat, was shown to interact with PPT1, inhibiting late-stage autophagy and blocking HCC cell proliferation [135]. Another investigation found that PPT1 is primarily expressed in HCC macrophages and that overexpression of PPT1^+^ macrophages is connected to poor patient prognosis [136]. These data indicate that targeting PPT1 may be a viable approach for treating HCC. Figure 6 describes a potential mechanism.

### Other digestive system diseases

Esophageal cancer (EC) is a kind of upper gastrointestinal cancers with high malignancy and poor prognosis [137]. Recent study discovered that FASN/PA-mediated Wnt3A (Cys77) palmitoylation promotes Wnt3A membrane localization and the translocation of β-catenin into the nucleus, further activating Wnt3A/β-catenin pathway and promoting the formation of EMT phenotype in esophageal squamous cell carcinoma [138]. Additionally, another study demonstrated that protein palmitoylation was affected by radiation in EC cell lines, which may be a viable approach for treating EC [139]. Beyond EC, palmitoylation also plays a crucial role in viral hepatitis. Studies have shown that palmitoylation is very important for hepatitis virus RNA replication and virion production. Liu et al. discovered that the ORF3 protein is palmitoylated at positions Cys18 and Cys21. Site-directed mutagenesis of these sites were unable to efficiently release ORF3 vesicles from hepatocytes, and inhibit binding with ANXA2, abolished hepatitis E virus replication [140]. The study between palmitoylation and other viral hepatitis (hepatitis B virus and hepatitis C virus) are described in the review by Wang [141]. Although palmitoylation is associated with multiple digestive diseases, the pancreatitis and gastroesophageal reflux disease remain underexplored.

## Potential applications

Recent research on palmitoylation in digestive system diseases has revealed its potential as a diagnostic biomarker and therapeutic target. Xu et al. reported that elevated ZDHHC3 levels in the liver tissues of patients with NASH are positively associated with the severity of the NASH phenotype, indicating ZDHHC3’s potential in assessing NASH severity [82]. GC, a highly malignant and rapidly progressing cancer of the digestive system with challenging treatment options, requires new biomarkers and therapeutic strategies [107]. Yan et al. evaluated the prognostic value of ZDHHC2 in GC and found that the 5-year survival rates between the low and high ZDHHC2 expression groups were significantly different (2.21% and 2.26%, respectively), suggesting that ZDHHC2 may serve as a potential prognostic

marker [108], consistent with the findings in HCC [109]. Additionally, Jiang et al. found that patients with HCC who exhibited high ZDHHC7 expression had poor clinical staging and prognosis, indicating that ZDHHC7 is a novel biomarker for HCC diagnosis [35]. Collectively, these findings indicate that palmitoyltransferases are promising diagnostic and prognostic markers for digestive tract cancers.

In addition to its diagnostic significance, palmitoylation is gradually recognized as a novel therapeutic target for digestive system diseases. However, only a few DHHC inhibitors have been identified to date. Lomitapide, a ZDHHC5 inhibitor, has demonstrated significant efficacy in reducing pancreatic tumor cell growth and proliferation, as validated both in vitro and in models [114]. Additionally, artemisinin, a ZDHHC6 inhibitor, effectively reduces N-Ras palmitoylation, thereby weakening downstream signaling pathways and providing a potential therapeutic strategy for N-Ras mutant cancers [142]. The therapeutic potential of depalmitoylation inhibition was further validated by Zhou et al.; hydroxychloroquine (HCQ), a PPT1 inhibitor, enhanced AEG-1 palmitoylation, subsequently inhibiting cell growth in HCC [33]. Palmitoylation exerts a significant effect on stabilizing the surface expression of PD-1 and PD-L1. For example, ZDHHC9-mediated PD-L1 palmitoylation has been indicated to stabilize PD-L1 protein levels in tumor cells, thereby promoting the efficacy of immune checkpoint inhibitors. PSP-S1, a novel compound, has exhibited potential by competitively inhibiting PD-L1 palmitoylation, thus preventing tumor immune evasion [38]. In summary, these dates emphasize the crucial value of palmitoylation in digestive system diseases, and targeting specific palmitoyltransferases or depalmitoylases could offer new strategies for the diagnosis and treatment of PC, HCC, and other digestive system diseases (Table 3).

**Table 3:** Potential applications of modulation of palmitoylation in digestive system diseases.

Inhibitors	Mechanisms	Disease	Relevant outcome	Refs.
2-BP	ZDHHC2	GC	Promoted anti-cancer immunity.	[25]
2-BP, CPP-S1	ZDHHC3	CRC	Modulated PD-L1 stability, overcoming immune evasion of tumor cells.	[38]
Lomitapide	ZDHHC5	PC	ZDHHC5 mediated palmitoylation on SSTR5 blocked by lomitapide, which contributed to an anti-proliferation effect.	[114]
ART	ZDHHC6	HCC	ART mediated ZDHHC6 inhibition diminished N-Ras palmitoylation, which disrupted the Golgi localization and signaling cascade of N-Ras.	[142]
MY-D-4	ZDHHC7	HCC	Blocked HCC cell proliferation.	[35]
2-BP	ZDHHC17	IBD	2-BP could suppress NLRP3-mediated colitis.	[20]
2-BP,CPPtat-Y1	ZDHHC20	PC	Impeded the progression of KRAS mutant PC.	[29]
2-BP	ZDHHC24	NASH	Ameliorated NASH and subsequent liver cancer by inhibiting AKT palmitoylation.	[34]
ML349	APT2	IBD	Relieved the symptoms of IBD.	[19]
HCQ	PPT1	HCC	HCQ enhances the degradation of AEG-1 and weakened the interaction between AEG-1 and SND1, which suppressed the proliferation of HCC.	[33]
GNS561/Ezurpimtrostat	PPT1	HCC	Showed antiproliferative and antitumor activity in animal and human models.	[135]
DC661	PPT1	HCC	Promoted the therapeutic efficacy of anti-PD-1 antibody in vivo.	[136]

This table illustrates the main inhibitors of ZDHHCs/APTs in digestive system diseases and their function.

## Conclusions and perspective

Palmitoylation is a type of lipid modification catalyzed by DHHC-PTAs and depalmitoylases that has a critical function in modulating protein localization, stability, and signal transduction. Palmitoylation has shown significant promise in the study of digestive system diseases, since it modulates key proteins and pathways. Notably, ZDHHC2, ZDHHC3, and ZDHHC7 have been recognized as potential biomarkers for GC, NAFLD, and HCC, respectively. Inhibitors targeting key ZDHHC enzymes, such as MY-D-4, lomitapide, and artemisinin have emerged as novel therapeutic agents. PA is involved in palmitoylation and modulates the expression of genes associated with FA metabolism, thereby affecting the progression of certain disorders. For example, SCD1, FASN, and ELOVL6 are associated with GC cell proliferation [143]. A major study showed that ACOX1 inhibits CRC progression by reprogramming PA metabolism [31]. Jeong et al. observed that elevated PA levels promote AKT palmitoylation, leading to liver cancer progression, suggesting that dietary PA levels should be closely monitored in patients with liver cancer. Developing highly efficient small-molecule drugs targeting key palmitoyltransferases or using PROTAC technology to directly degrade these enzymes is an innovative strategy for precise treatment of digestive system diseases [144].

Although significant advancements in comprehending palmitoylation in malignance research, its role in benign digestive system conditions, such as pancreatitis and gastroesophageal reflux disease, remains underexplored. A total of 23 ZDHHC enzymes mediate protein palmitoylation, each with multiple substrates. The specific substrates corresponding to each class of enzymes have not yet been fully defined, and the mechanisms of enzyme-substrate specificity remain unclear. For specific protein substrates, only designated cysteine residues undergo S-palmitoylation; however, the same substrate can be catalyzed by multiple ZDHHC enzymes. Notably, same substrate is palmitoyl by the same ZDHHC enzymes at different Cys sites, and the functions of the substrate are also different, such as NLRP3. A recent review by Zhang et al. provides a comprehensive overview of these discrepancies, highlighting potential reasons for the divergent results [145]. Addressing these challenges will deepen the understanding of palmitoylation. Currently, the methods for detecting palmitoylation primarily include acyl-biotin exchange (ABE), biorthogonal detection, acyl-RAC, and liquid chromatography-mass spectrometry-based proteomics [146]. A novel tool, SwissKASH, was recently developed for visualizing palmitoylation. Advancements in these techniques have provided a solid foundation for understanding the mechanisms and molecular principles underlying palmitoylation [147].

Despite significant advancements in the study of palmitoylation, several challenges remain. First, the reversible nature of palmitoylation complicates the precise detection and characterization of this modification in vivo. Traditional methods, such as mass spectrometry, can identify and quantify palmitoylation states, but fail to capture real-time dynamic changes. Second, the hydrophobic nature of palmitoylation makes it difficult to solubilize and purify the modified proteins, further complicating research. Another major challenge is the lack of specific antibodies or probes for the detection of palmitoylation. Existing probes often struggle to distinguish between different palmitoylated proteins, making precise cellular localization difficult.

Future research should focus on several key areas as proteomics technology continues to advance, particularly with the development of time-resolved mass spectrometry that can precisely capture dynamic changes. The development of highly specific palmitoylation antibodies and probes will also facilitate the real-time imaging of live cells or tissues, facilitating the capture of dynamic palmitoylation changes. Future investigations should focus on the following aspects: (1) investigating the functions of ZDHHC enzymes and depalmitoylases and clarifying the substrate specificity of each ZDHHC family member and their specific roles in different cell types and tissues; (2) identifying more therapeutic strategies targeting palmitoylation by exploring the potential of palmitoylation as a drug target and creating chemical probes and small molecules that regulate the activity of specific ZDHHCs or depalmitoylases; (3) utilizing new technologies to study palmitoylation dynamics and employing advanced imaging and mass spectrometry techniques to monitor palmitoylation changes in cells and their alterations in pathological states.

Palmitoylation provides promising insights into the pathogenesis of digestive system diseases, and opens novel avenues for clinical diagnosis and treatment. In diagnostics, abnormal expression or dysfunction of proteins related to palmitoylation can serve as early biomarkers for disease detection and monitoring. Drugs targeting palmitoylation hold significant promise for treatment. For instance, modulating the activity of specific ZDHHC enzymes or depalmitoylases can restore abnormal signaling pathways in diseased states, thereby effectively halting disease progression. Palmitoylation-targeted immunotherapy has emerged as a new strategy for treating cancer and inflammatory diseases. The development of these therapies will help improve patient outcomes and reduce the impact of diseases on the quality of life.
